# Cancer‐Derived Extracellular Vesicle ITGB2 Promotes the Progression of Triple‐Negative Breast Cancer via the Activation of Cancer‐Associated Fibroblasts

**DOI:** 10.1002/gch2.202400235

**Published:** 2025-01-23

**Authors:** Jingjing Fan, Tong Sha, Binlin Ma

**Affiliations:** ^1^ Xinjiang Key Laboratory of Oncology The Affiliated Cancer Hospital of Xinjiang Medical University Urumqi 830011 China; ^2^ The Clinical Medical Research Center of Breast and Thyroid Tumor in Xinjiang Urumqi 830011 China

**Keywords:** cancer‐associated fibroblast, extracellular vesicle, integrin beta 2, triple‐negative breast cancer, tumor microenvironment

## Abstract

Breast cancer is the most prevalent cancer and a leading cause of death among women globally, posing a significant public health challenge. Triple‐negative breast cancer (TNBC), an aggressive subtype accounting for 15–20% of all breast cancers, lacks targeted therapies due to the absence of hormone receptors and HER2, resulting in poor prognosis and high recurrence rates. This study investigates the role of cancer‐derived extracellular vesicle (EV) integrin beta‐2 (ITGB2) in TNBC progression. These findings reveal that ITGB2 is significantly overexpressed in TNBC tissues and serum EVs, correlating with advanced tumor stages and poor patient survival. ITGB2 enhances TNBC progression by activating cancer‐associated fibroblasts (CAFs) within the tumor microenvironment, promoting tumor growth, migration, and invasion. Mechanistic studies demonstrate that EV ITGB2 facilitates CAF activation, driving tumor‐stroma interactions that support TNBC progression. These results highlight ITGB2 as a potential biomarker and therapeutic target in TNBC, emphasizing the need for novel interventions to combat this challenging breast cancer subtype.

## Graphical abstract

This study investigates the role of cancer‐derived extracellular vesicle (EV) integrin beta‐2 (ITGB2) in the progression of triple‐negative breast cancer (TNBC). ITGB2 is upregulated in TNBC tissues and serum EVs, correlating with poor prognosis. The study reveals that ITGB2 facilitates the activation of cancer‐associated fibroblasts (CAFs), enhancing tumor progression and providing potential therapeutic implications for targeting ITGB2 in TNBC treatment.

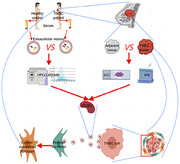

## 1 Introduction

Breast cancer is the most common cancer and the leading cause of morbidity and mortality among women worldwide.^[^ ^1^ ^]^ Triple‐negative breast cancer (TNBC) is the most aggressive subtype of breast cancer, accounting for 15–20% of this malignancy.^[^ ^2^, ^3^ ^]^ The lack of hormone receptor (HR) and human epidermal growth factor receptor 2 (HER2) expression in TNBC means that conventional therapies targeting these receptors, such as hormone therapy and HER2‐targeted therapies, are not effective. However, TNBC still presents potential for targeted therapies, such as those targeting other pathways or immune checkpoints. Currently, the standard non‐surgical treatment for TNBC involves non‐specific chemotherapeutic agents, which are often ineffective. Consequently, TNBC patients have high rates of recurrence, distant metastasis, and mortality.^[^ ^4^, ^5^, ^6^ ^]^ There is an urgent need for new therapeutic approaches, preferably targeted therapies. To achieve this, it is crucial to understand the mechanisms driving TNBC progression.

Tumors develop within a dynamic ecosystem known as the tumor microenvironment (TME). The TME is composed of tumor stroma, blood vessels, infiltrating immune cells, fibroblasts, and other cell types. The cellular and molecular components of the TME interact continuously with tumor cells, influencing tumor proliferation, immune evasion, and metastasis.^[^ ^7^, ^8^, ^9^ ^]^ For instance, cancer‐associated fibroblasts (CAFs) are major components of the tumor stroma, capable of producing various cytokines, growth factors, and extracellular matrix components, thereby forming the tumor stroma and affecting the behavior of tumor cells.^[^ ^10^ ^]^ Conversely, tumor cells secrete extracellular vesicles (EVs) that deliver tumor‐derived proteins and other cellular components to the tumor stroma.^[^ ^11^ ^]^ Tumor‐derived EVs can either inhibit or promote tumor development, depending on their cargo. Increasing evidence suggests that EVs from breast cancer cells contribute to tumor proliferation and metastasis. However, the detailed mechanisms underlying these effects remain largely unclear.

Integrins are a large family of cell adhesion receptors composed of α and β transmembrane glycoproteins. As master regulators of cell adhesion and migration, integrins are implicated in almost every step of cancer progression.^[^ ^12^, ^13^ ^]^ Interestingly, recent studies have discovered that integrins carried in tumor EVs can direct the formation of pre‐metastatic niches by binding to organ‐specific markers.^[^ ^14^, ^15^ ^]^ Integrin beta 2 (ITGB2) is a beta chain protein of the integrin family that is upregulated in human TNBC tissues and cells, with its expression associated with tumor stage, lymph node metastasis, and poor prognosis.^[^ ^16^, ^17^, ^18^, ^19^ ^]^ However, the functional role of ITGB2 in TNBC progression remains unclear. In this study, we evaluated the expression of ITGB2 in human TNBC tissues, cells, and serum EVs, as well as the relationship between tumor ITGB2 expression and disease progression and prognosis. Additionally, we investigated the effect of ITGB2 overexpression on in vivo TNBC tumorigenesis. Mechanistically, we explored the role of tumor EV ITGB2 in activating CAFs both in vitro and in vivo.

## 2 Results

### 2.1 High Expression of ITGB2 in Serum EVs and Tissue of TNBC Patients

**Figure 1:**
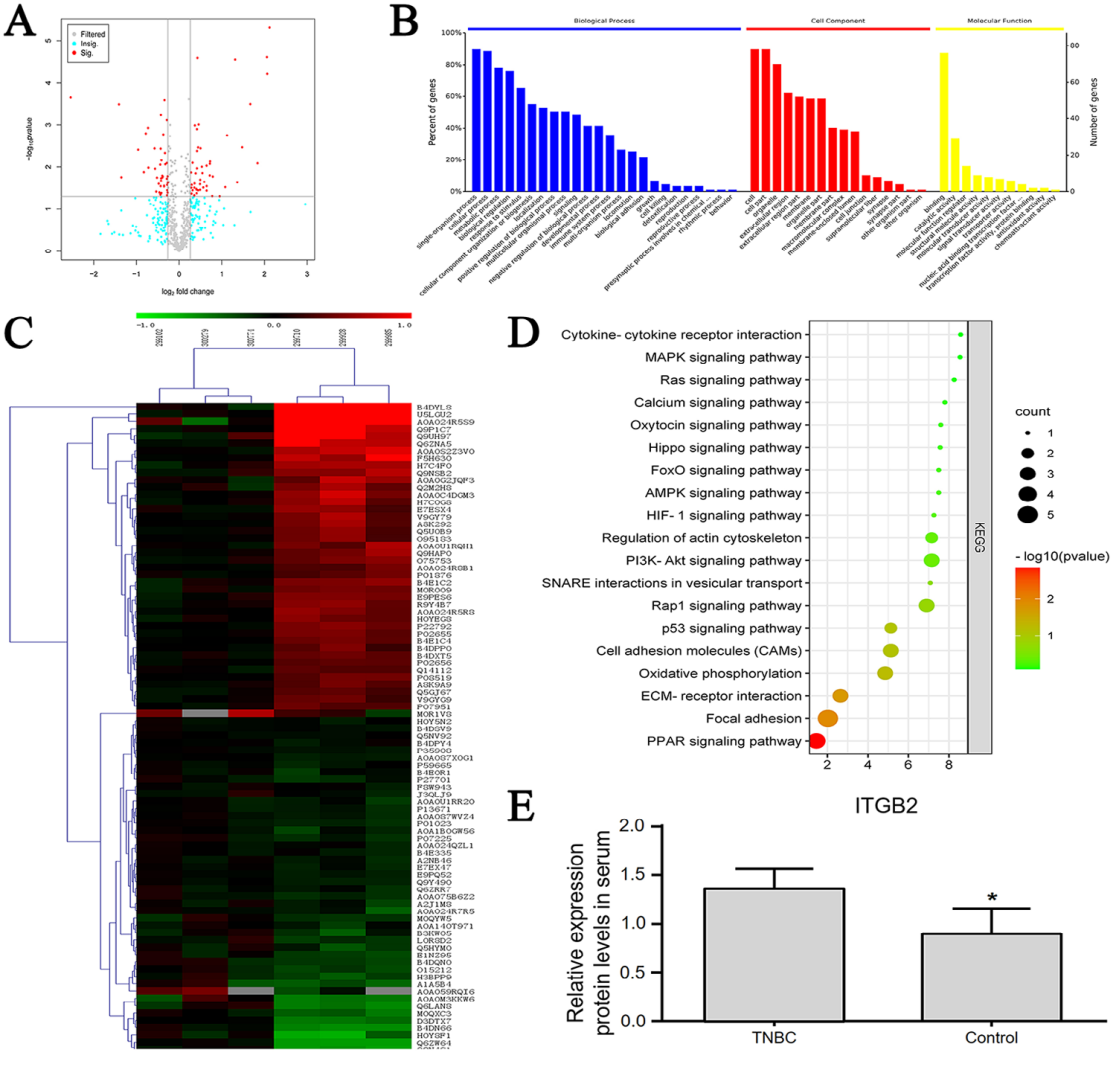
Proteomic analysis of serum EVs from TNBC patients. A) Scatter plot of DEPs in TNBC serum EVs compared to healthy controls, identified via HPLC‐MS/MS. Red dots indicate proteins with > twofold change and statistically significant difference (*P* < 0.05); green dots indicate proteins with > twofold change but not significant (*P* > 0.05); gray dots indicate proteins with <twofold change and not significant. B) GO enrichment analysis of DEPs. C) Heatmap of DEPs, showing hierarchical clustering of protein expression. D) KEGG pathway enrichment analysis of DEPs, highlighting pathways involved in cell adhesion, migration, and immune responses. E) ITGB2 expression levels in TNBC serum EVs compared to controls, measured by proteomic analysis. Data are expressed as mean ± SD (*n* = 3), *P < 0.05 versus control.

**Figure 2:**
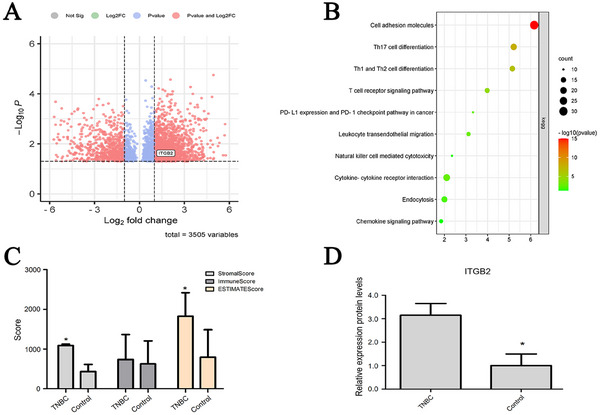
Meta‐analysis of gene expression and immune cell profiling in TNBC tissues. A) Scatter plot showing DEGs in TNBC tissues compared to adjacent normal tissues, based on GEO database analysis. Red dots represent genes with >twofold change and statistically significant difference (*P* < 0.05); blue dots represent non‐significant changes. B) GO and KEGG enrichment analysis of DEGs, highlighting pathways related to cell adhesion and immune regulation. C) Proportions of stromal and immune cell populations in TNBC and adjacent tissues, estimated using CIBERSORTx. D) Expression levels of ITGB2 in TNBC tissues compared to adjacent tissues. Data are expressed as mean ± SD (*n* = 93), *P < 0.05 versus control.

**Figure 3:**
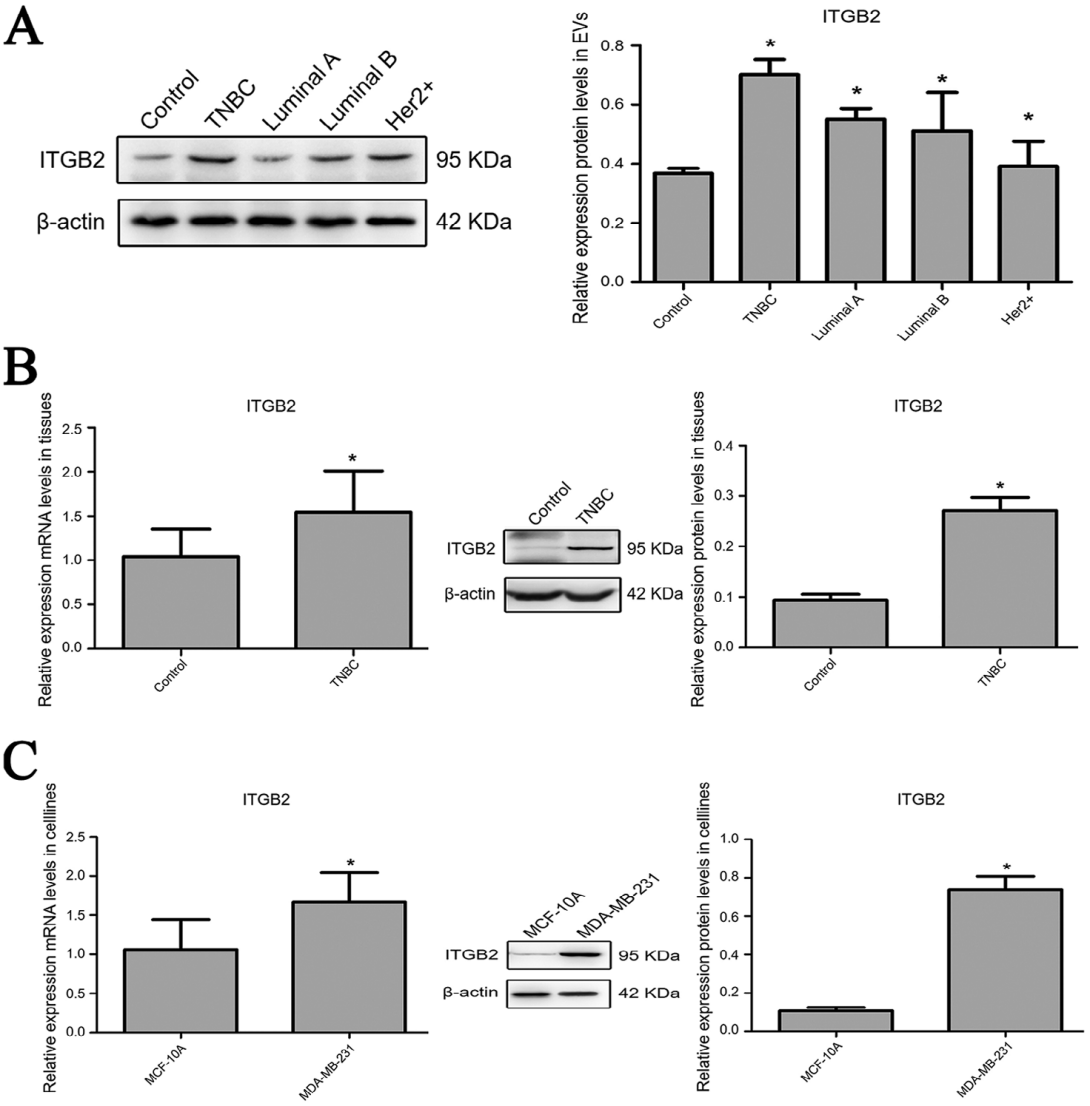
ITGB2 expression in TNBC tissues, serum EVs, and cell lines. A) Western blot analysis of ITGB2 levels in serum EVs from TNBC, Luminal A, Luminal B, Her2+ breast cancer patients, and healthy controls. Data are expressed as mean ± SD (*n* = 3 per group), *P < 0.05 versus control. B) ITGB2 expression in TNBC tissues versus adjacent normal tissues, assessed by western blot and qRT‐PCR (*n* = 30), *P < 0.05 versus control. C) ITGB2 expression in MDA‐MB‐231 TNBC cells compared to MCF‐10A normal mammary epithelial cells, measured by western blot and qRT‐PCR (*n* = 3), *P < 0.05 versus MCF‐10A.

Initially, we characterized the serum EVs from TNBC patients and healthy controls using NTA. Compared to the control group, EVs from TNBC patients displayed larger particle sizes and higher particle density. Subsequent proteomic analysis of these serum EVs using HPLC‐MS/MS identified 90 differentially expressed proteins (DEPs) in TNBC EVs compared to controls (fold change > 2, *P* < 0.05). Of these, 48 proteins were upregulated, and 42 were downregulated in TNBC EVs (**Figure** **1**A). GO and KEGG pathway enrichment analyses revealed that these DEPs are primarily involved in pathways controlling cell adhesion, migration, homeostasis, oxidative stress, and immune responses (Figure 1B–D). ITGB2, a cell adhesion receptor, was found to be upregulated in human TNBC, with its expression in TNBC serum EVs being 1.33 times higher than that in controls (Figure 1E). Additionally, we performed a meta‐analysis using TNBC sequencing data from the GEO database (GSE176078, GSE177043, and GSE58135), identifying 3505 differentially expressed genes (DEGs) in human TNBC tissues compared to paired adjacent tissues (fold change > 2, *P* < 0.05) (**Figure** **2**A). GO and KEGG pathway enrichment analyses using DAVID indicated that these DEGs are predominantly involved in 45 pathways, with the highest enrichment scores in cell adhesion and immune regulation pathways (Figure 2B). Notably, ITGB2 expression in TNBC tissues was 3.1 times higher than that in adjacent control tissues (Figure 2D). CIBERSORTx, a tool used to estimate the abundance of cell types within mixed cell populations from large‐scale gene expression data,^[^ ^12^ ^]^ showed that TNBC tissues had a higher proportion of stromal and immune cell populations compared to controls (Figure 2C). These results are consistent with previous findings on stromal cell activation and immune cell infiltration during TNBC progression. Given the crucial role of integrins in cancer progression, the upregulation of ITGB2 in TNBC tissues and serum EVs is likely associated with TNBC development. To further investigate this molecule, we used Western blot analysis to measure its levels in serum EVs from TNBC patients and other breast cancer subtypes, including Luminal A, Luminal B, and Her2+. The results showed elevated expression levels across all breast cancer subtypes, with the highest expression observed in TNBC patients (0.701 ± 0.052 vs 0.368 ± 0.017, *P* = 0.013; **Figure** **3**A). Subsequent Western blot analysis confirmed the upregulation of ITGB2 in human TNBC tissues compared to adjacent normal tissues (0.271 ± 0.026 vs 0.094 ± 0.011, *P* = 0.000; Figure 3B) and in MDA‐MB‐231 human TNBC cells compared to MCF‐10A normal mammary epithelial cells (0.738 ± 0.070 vs 0.107 ± 0.018, *P* = 0.000; Figure 3C). Furthermore, qRT‐PCR also confirmed ITGB2 overexpression in human TNBC tissues and cells (Figure 3B,C).

### 2.2 Association between Tumoral ITGB2 Levels and Survival in TNBC Patients

**Figure 4:**
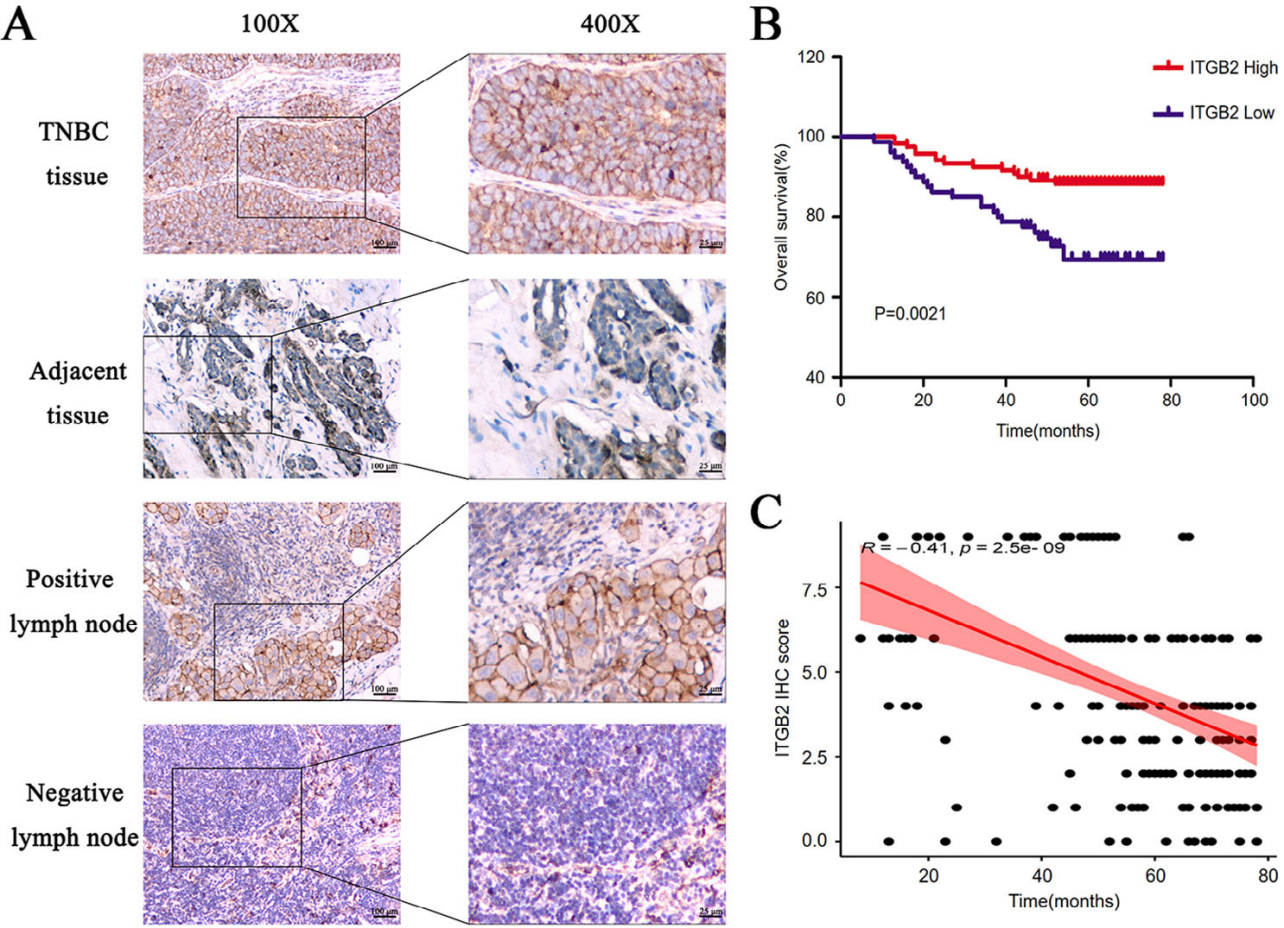
ITGB2 expression correlates with clinicopathological features and survival in TNBC patients. A) Representative IHC staining of ITGB2 in TNBC tissues and adjacent normal tissues. B) Kaplan‐Meier survival analysis comparing patients with high ITGB2 expression (*n* = 80) and low ITGB2 expression (*n* = 120) in tumor tissues. *P < 0.05. C) Pearson correlation between ITGB2 IHC scores and overall survival in TNBC patients (*R* = ‐0.41, *P* = 0.0021).

To assess the relationship between ITGB2 expression and patient survival, we determined ITGB2 protein levels in 200 TNBC patients using IHC staining. The demographic and clinicopathological characteristics of the patients are shown in **Table** **1**. ITGB2 expression was significantly associated with tumor size, tumor stage, grade, lymph node metastasis, and vascular invasion. Consistent with Western blot results, higher ITGB2 IHC staining signals were detected in tumor tissues compared to adjacent normal tissues (4.72 ± 1.49 vs 3.07 ± 2.52, *P* = 0.020; **Figure** **4**A). Based on the total ITGB2 staining score in tumor tissues, patients were divided into ITGB2 low expression (*n* = 120) and high expression groups (*n* = 80). Kaplan‐Meier survival analysis showed that the 5‐year survival rate was lower in the ITGB2 high expression group compared to the low expression group (45 months vs 58 months, *P* = 0.002; Figure 4B). Moreover, Pearson correlation analysis revealed a negative correlation between tumor ITGB2 scores and survival time (*R* = ‐0.41, *P* = 0.0021; Figure 4C).

**Table 1:** Relationship between ITGB2 expression and clinicopathological characteristics in TNBC patients.

Characteristics	Number	ITGB2 expression(x±s)	*χ* ^2^	*P*‐value
Age			5.429	0.066
≤35	18	3.92±1.89		
35<age≤55	136	3.47±2.33		
55<	46	2.62±2.23		
Menopausal status			1.312	0.204
Premenopausal	117	3.52±2.31		
Postmenopausal	83	3.02±2.26		
Tumor stage			3.925	**0.019**
I	51	2.45±2.04		
II	112	3.54±2.21		
III	37	3.82±2.62		
Lymph node metastasis			2.070	**0.038**
NO+N1	161	3.50±1.96		
N2+N3	40	4.38±2.70		
Tumor size (cm)			3.763	**0.013**
*T*≤2	80	2.98±2.45		
2<*T*≤5	108	3.49±2.22		
5<*T*	12	3.89±1.90		
Histological grade			2.108	**0.022**
Grade 2	75	2.79±2.18		
Grade 3	114	3.58±2.18		
ki‐67(%)			4.578	**<0.001**
≤50	49	2.86±1.59		
>50	151	4.78±2.29		
Vascular invasion			0.212	**<0.001**
No	169	3.37±2.38		
Yes	31	4.40±2.51		

Bold value in Table 1 indicates that this *p*‐value is statistically significant.

### 2.3 ITGB2 in TNBC EVs Promotes Fibroblast‐to‐CAF Transition and CAF Activation

**Figure 5:**
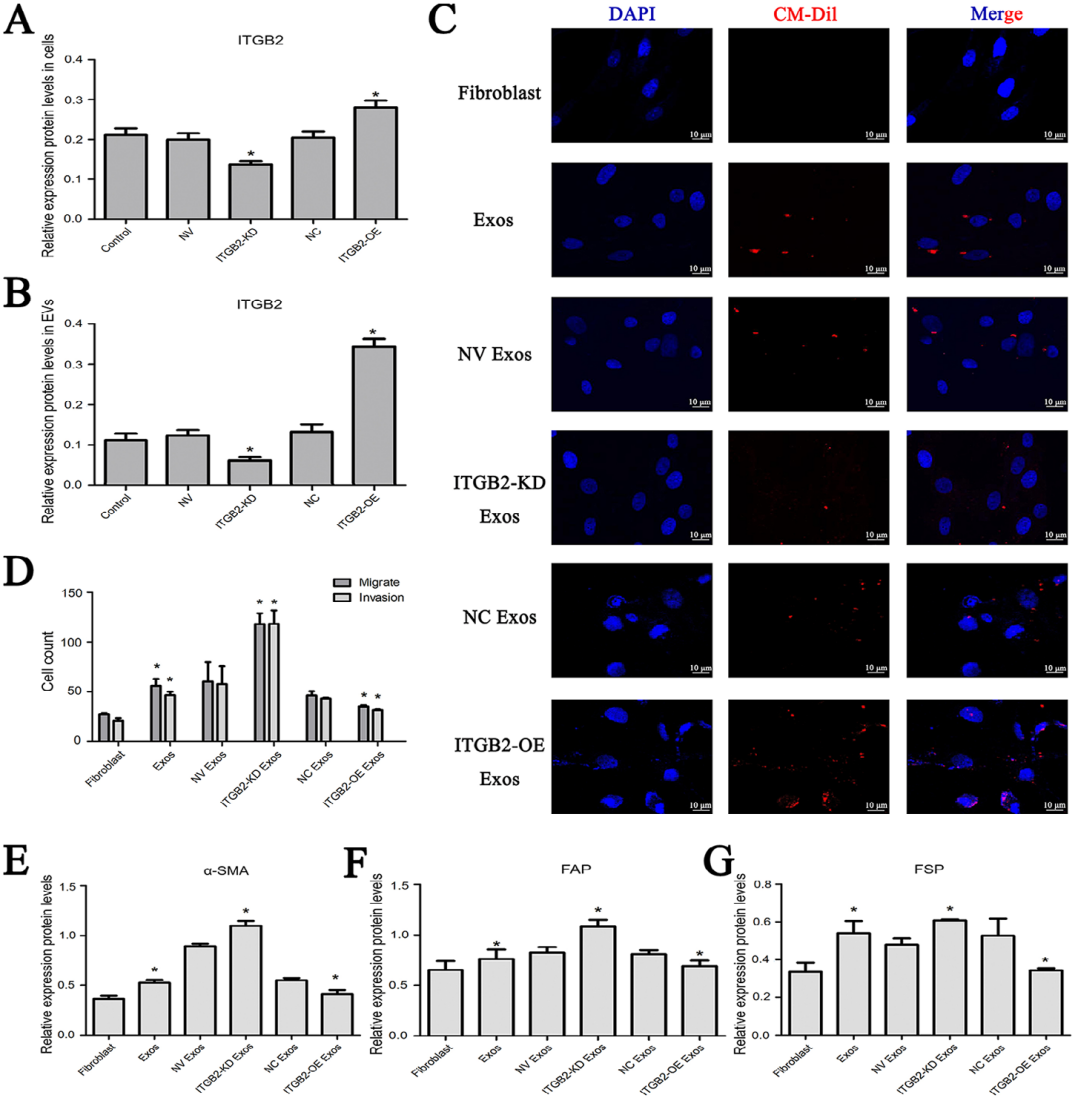
ITGB2 in TNBC EVs promotes fibroblast‐to‐CAF transition and activation. A) Western blot analysis of ITGB2 expression in wild‐type (Control), ITGB2‐overexpressing (ITGB2‐OE), and ITGB2‐knockdown (ITGB2‐KD) MDA‐MB‐231 cells. B) ITGB2 levels in EVs derived from wild‐type, ITGB2‐OE, and ITGB2‐KD MDA‐MB‐231 cells, measured by western blot. C) Confocal fluorescence microscopy showing the uptake of ITGB2‐labeled EVs by fibroblasts after 24 h of incubation. D) Migration and invasion assays of fibroblasts treated with EVs from ITGB2‐OE, ITGB2‐KD, or control cells. E–G) Western blot analysis of α‐SMA, FAP, and FSP levels in fibroblasts treated with ITGB2‐expressing or ITGB2‐knockdown EVs. Data are expressed as mean ± SD, *P < 0.05 versus control.

CAFs are key components of TME that regulate tumor progression. CAFs primarily originate from local fibroblasts. The transition from fibroblasts to CAFs involves cell proliferation and migration, while CAF activation is marked by the production of α‐SMA, FAP, and FSP.^[^ ^20^, ^21^, ^22^ ^]^ In this study, To investigate the role of ITGB2 in these effects, we transfected MDA‐MB‐231 cells with a lentiviral system expressing ITGB2 or ITGB2 shRNA. Overexpression and knockdown of ITGB2 both in TNBC cells (**Figure** **5**A) and TNBC cell derived EVs (Figure 5B) were confirmed by Western blot analysis. MCR‐5 cells were treated with EVs from wild‐type control and transfected MDA‐MB‐231 cells for 24 h. We found that EVs expressing ITGB2 enhanced MCR‐5 cell migration and invasion (Figure 5D) and increased α‐SMA, FAP, and FSP production compared to control EVs, whereas EVs with ITGB2 knockdown had the opposite effect (Figure 5E–G). These results indicate that ITGB2 in TNBC EVs promotes fibroblast‐to‐CAF transition and CAF activation.

### 2.4 ITGB2 in TNBC EVs Mediates Fibroblast Uptake of EVs

To explore the mechanism by which ITGB2 in MDA‐MB‐231 EVs promotes fibroblast‐to‐CAF transition and CAF activation, we used confocal fluorescence microscopy to assess the uptake of MDA‐MB‐231 EVs by MCR‐5 cells. EVs were labeled with the CM‐Dil dye (red) for fluorescence detection. Significant uptake was detected after 24 h of incubation (Figure 5C). Compared to control EVs, MCR‐5 cells showed more pronounced uptake of ITGB2‐expressing EVs, while uptake of ITGB2‐knockdown EVs was nearly undetectable. These results suggest that ITGB2 in tumor EVs mediates fibroblast uptake of these EVs in the TNBC TME, potentially through interactions with cell surface receptors on recipient cells. The subsequent release of EV cargo may affect signaling, migration, and protein production in recipient fibroblasts, promoting their transition to and activation as CAFs.

### 2.5 Tumor ITGB2 Promotes TNBC Tumorigenesis in Nude Mice

**Figure 6:**
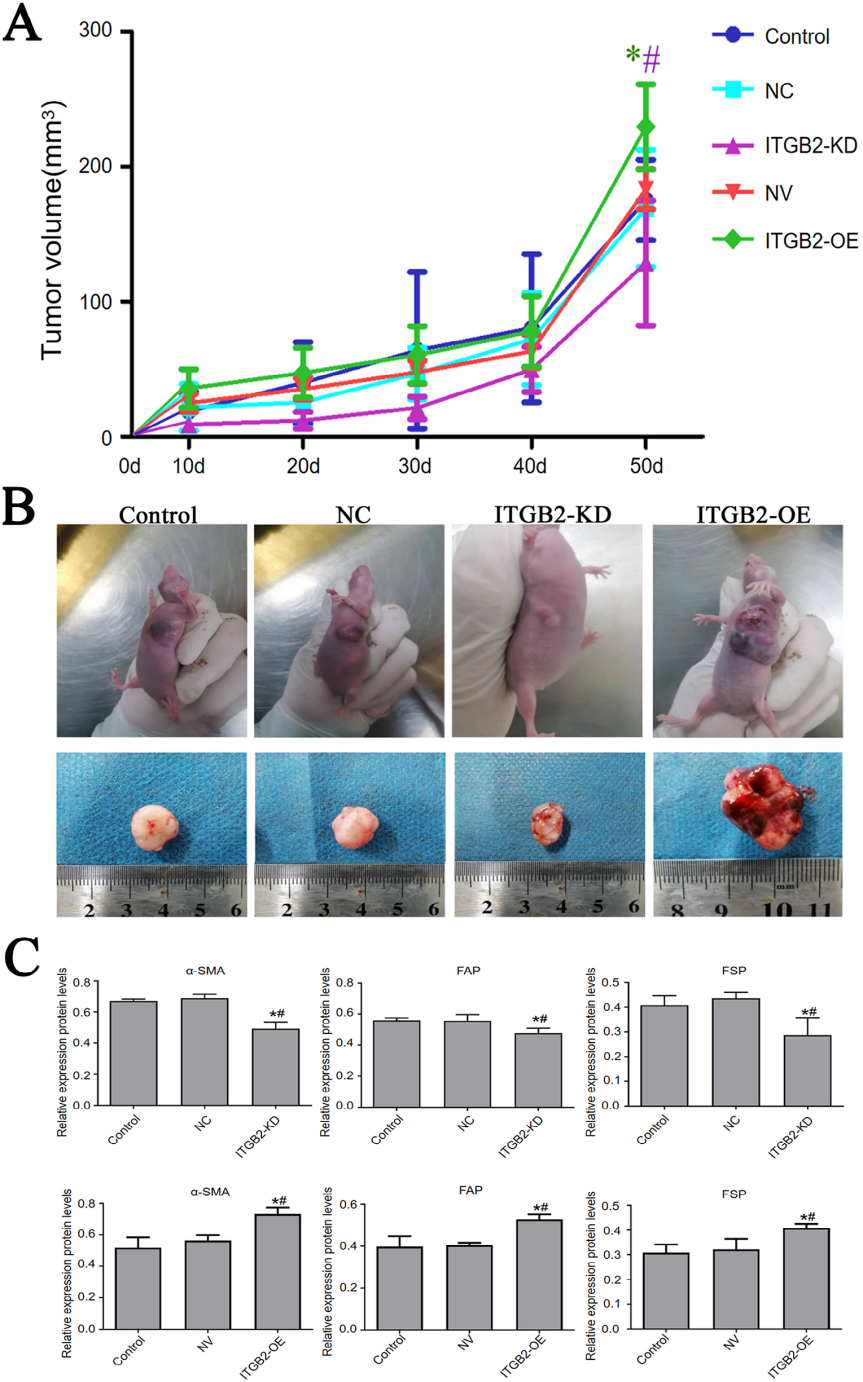
ITGB2 promotes TNBC tumorigenesis and CAF activation in vivo. A) Tumor volumes in nude mice inoculated with wild‐type, ITGB2‐overexpressing, or ITGB2‐knockdown MDA‐MB‐231 cells, measured on day 50 post‐inoculation (*n* = 6). B) Representative images of tumors excised from nude mice in each group. C) Western blot analysis of CAF activation markers (α‐SMA, FAP, FSP) in tumor tissues from each group. Data are expressed as mean ± SD, *P < 0.05 versus control, #*P* < 0.01 versus knockdown group.

Tumor volume in a human TNBC nude mouse model was measured in the 50th day post‐inoculation. Tumors generated by MDA‐MB‐231 cells expressing ITGB2 grew significantly faster than those generated by control cells, while tumors from ITGB2 knockdown cells grew more slowly (**Figure** **6**A). Consistent with these data, tumors in the ITGB2‐OE group were significantly larger than those in the Control and NC groups, while tumors in the ITGB2‐shRNA group were significantly smaller (Figure 6B). These in vivo results suggest that tumor ITGB2 promotes TNBC tumorigenesis.

### 2.6 Tumor ITGB2 Promotes CAF Activation in TNBC

**Figure 7:**
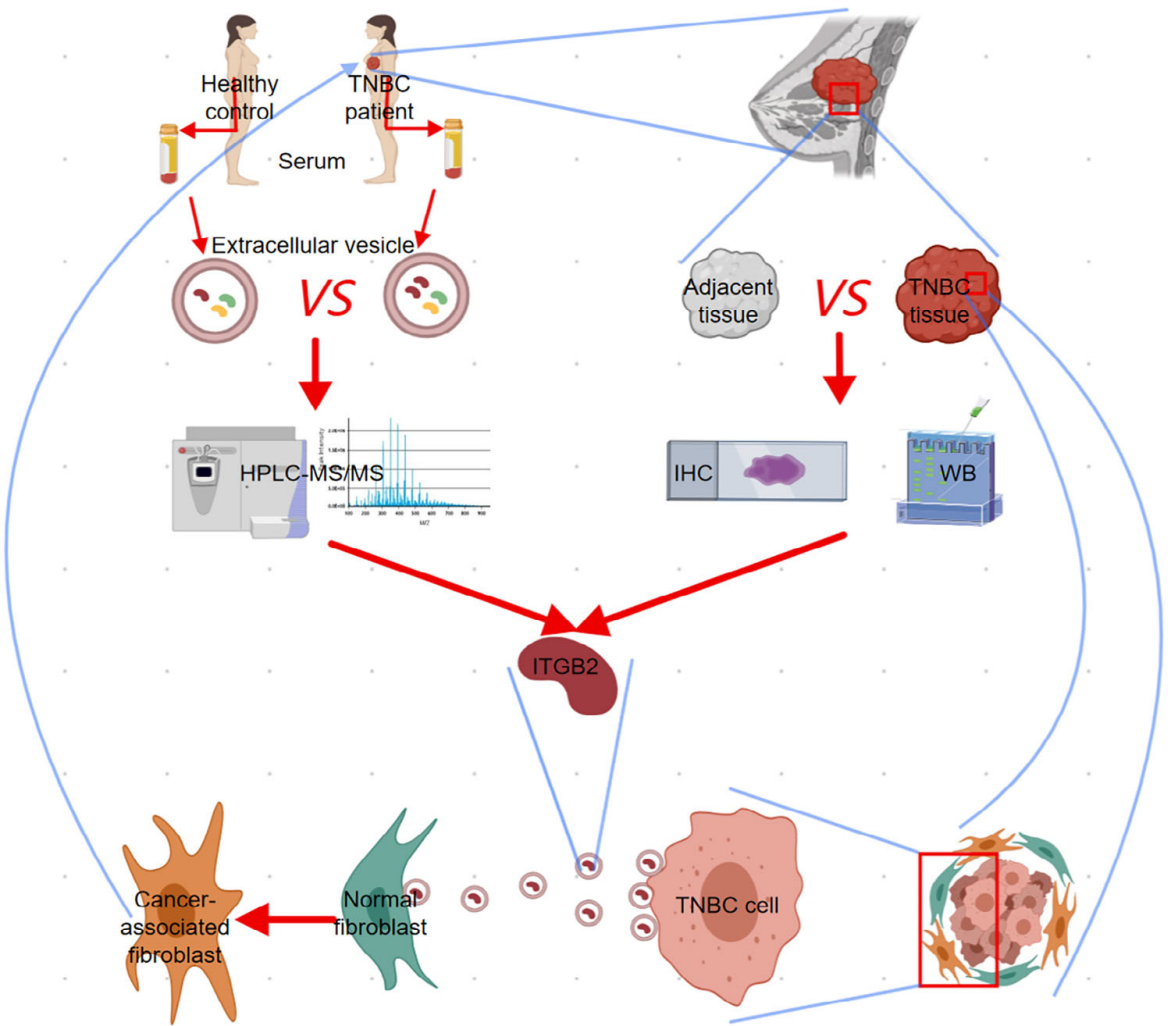
Graphical summary of the study: ITGB2 in TNBC progression and tumor microenvironment remodeling. Tumor‐derived EVs enriched in ITGB2 are secreted by TNBC cells. ITGB2 facilitates the uptake of EVs by fibroblasts, promoting their transition into CAFs through the upregulation of activation markers (α‐SMA, FAP, and FSP). Activated CAFs, in turn, enhance tumor‐stroma interactions, fostering tumor growth, migration, and invasion. Additionally, ITGB2 overexpression correlates with poorer patient prognosis and higher tumor aggressiveness. These findings highlight ITGB2 as a key mediator of TNBC progression and a potential target for therapeutic intervention.

To evaluate the effect of tumoral ITGB2 on CAFs within the TME, we measured protein levels of CAF activation markers α‐SMA, FAP, and FSP in tumor tissues using Western blot analysis. The levels of these three markers were significantly elevated in tumors expressing ITGB2 compared to controls, indicating enhanced CAF activation within the tumor tissues. In contrast, marker levels were lower in ITGB2‐knockdown tumors than in controls, indicating reduced CAF activation within the TME (Figure 6C). Collectively, these data indicate that ITGB2‐enriched EVs from TNBC cells actively promote fibroblast‐to‐CAF transition and enhance tumor‐stroma interactions, as illustrated in **Figure** **7**.

## 3 Discussion

In this study, we explored the role of ITGB2 in TNBC, focusing on its expression in tumor tissues, serum EVs, and its influence on tumor progression and the TME. Our results revealed that ITGB2 is significantly overexpressed in TNBC tissues and serum EVs compared to matched adjacent normal tissues. This overexpression is strongly correlated with poorer overall survival in TNBC patients, suggesting that ITGB2 could serve as a prognostic biomarker for this aggressive breast cancer subtype.

ITGB2 is overexpressed in several cancers, and its expression levels are associated with disease progression and poor prognosis. However, it remains unclear whether ITGB2 functionally contributes to the development of TNBC. In this study, we detected elevated levels of ITGB2 in tumor tissues, serum EVs from TNBC patients, and human TNBC cell lines. Consistent with previous findings,^[^ ^23^, ^24^, ^25^ ^]^ we found that tumor ITGB2 was negatively correlated with overall survival in TNBC patients. In a human TNBC nude mouse model, tumors expressing ITGB2 grew faster than those in the control group, while tumors lacking ITGB2 exhibited inhibited growth. These findings support the potential of ITGB2 as a therapeutic target for TNBC.

ITGB2 plays a critical role in immune response and cell adhesion, which are two fundamental cellular events controlling tumor initiation, progression, and metastasis.^[^ ^26^, ^27^ ^]^ Elevated levels of ITGB2 have been detected in various malignancies, including osteosarcoma,^[^ ^28^ ^]^ colorectal cancer,^[^ ^29^ ^]^ and glioma.^[^ ^30^ ^]^ Furthermore, ITGB2 expression is associated with poor clinical outcomes in glioma,^[^ ^31^ ^]^ high‐grade ovarian cancer,^[^ ^32^ ^]^ and acute myeloid leukemia.^[^ ^33^, ^34^, ^35^ ^]^ As a leukocyte adhesion receptor, ITGB2 forms a complex with its α‐chain partners to regulate the differentiation, migration, and activation of T cells, B cells, macrophages, neutrophils, and NK cells.^[^ ^36^, ^37^ ^]^ Mutations in the human ITGB2 gene can lead to leukocyte adhesion deficiency‐1, a rare and often fatal disorder characterized by severe immunodeficiency.^[^ ^38^ ^]^ In colorectal cancer, ITGB2 expression is associated with poor prognosis and increased infiltration of myeloid‐derived suppressor cells.^[^ ^39^ ^]^ In glioma, ITGB2 is linked to immune activation status and response to immunotherapy.^[^ ^40^ ^]^ In acute myeloid leukemia, ITGB2 significantly influences the infiltration of immune cells into the tumor stroma.^[^ ^41^ ^]^ Although most studies on ITGB2 in cancer have focused on its immunoregulatory functions, ITGB2 can also influence tumor growth and spread by mediating signaling between tumor cells and between tumor cells and the TME. For instance, in vitro, ITGB2‐deficient colorectal cancer cells show reduced proliferation, adhesion, and migration, and they fail to activate liver sinusoidal endothelial cells, which are essential for creating a TME conducive to liver metastasis. Consequently, tumor growth and liver metastasis are significantly reduced in vivo.^[^ ^42^ ^]^ Similar to findings in colorectal cancer cells, overexpression of ITGB2 in breast cancer cells also promotes cell migration and invasion in vitro.^[^ ^43^ ^]^

In this study, we found that EVs derived from TNBC cells could promote the transition of fibroblasts into CAFs and activate CAFs in an in vitro co‐culture system. Importantly, EVs enriched in ITGB2 were more readily phagocytosed by fibroblasts, thereby having a more profound impact on CAF transition and activation. As a cell adhesion receptor, ITGB2 likely facilitates the uptake of EVs by fibroblasts through interactions with surface receptors on recipient cells. Interestingly, a recent study found that CAFs in oral squamous cell carcinoma (OSCC) exhibit higher ITGB2 expression compared to matched normal fibroblasts.^[^ ^44^ ^]^ Additionally, conditioned media from ITGB2‐expressing CAFs was more effective in promoting OSCC cell proliferation than that from wild‐type controls. Mechanistically, ITGB2 upregulates the PI3K/AKT/mTOR pathway and activates glycolytic activity in CAFs.^[^ ^45^, ^46^, ^47^, ^48^, ^49^ ^]^ Taken together, these findings provide preliminary evidence that ITGB2 mediates crosstalk between tumor cells and CAFs, creating a TME that favors tumor progression. While this study primarily investigated how ITGB2 in TNBC cells influences CAFs, we speculate that ITGB2 may mediate communication between tumor cells and other TME components through various mechanisms, which warrant further investigation.

Due to high levels of tumor‐infiltrating lymphocytes and PD‐L1 expression, TNBC is the breast cancer subtype most amenable to immunotherapy.^[^ ^50^ ^]^ Indeed, PD‐1 blockers have been approved as first‐line treatment for PD‐L1‐positive metastatic TNBC patients, and new immunotherapeutic strategies are actively being developed.^[^ ^51^, ^52^, ^53^, ^54^ ^]^ TNBC is a heterogeneous disease with diverse immunogenicity. Identifying predictive biomarkers for patient selection is crucial for making immunotherapy a more personalized approach and expanding clinical benefits. ITGB2, reported as a master regulator of T lymphocytes and other immune cells, has been shown to predict immunotherapy response in glioma patients.^[^ ^40^ ^]^ However, there are few reports on the immunoregulatory function of ITGB2 in TNBC. In this study, bioinformatics analysis of the GEO database revealed numerous dysregulated immune regulatory genes in TNBC, with ITGB2 showing a 3.1‐fold upregulation. Additionally, CIBERSORTx analysis indicated a higher abundance of immune cell populations in TNBC tissues compared to adjacent normal tissues. Whether ITGB2 proliferation or depletion alters the immunogenicity of TNBC and whether ITGB2 can serve as a predictive marker for TNBC immunotherapy response remain to be seen. Our results suggest that ITGB2 is a key factor in the activation of CAFs, but it is likely that other cargoes within the EVs act in a complementary and synergistic manner to promote the fibroblast‐to‐CAF transition. ITGB2 may contribute to the process, but it is important to acknowledge the complexity of the tumor microenvironment, where multiple signals and molecules interact to drive CAF activation.

While our study provides valuable insights into the role of ITGB2 in TNBC, there are several limitations to consider. Firstly, in this study, we isolated EVs from serum using the exoEasy Maxi Kit (Qiagen), a widely used method for EV extraction. However, it is important to note that lipoproteins in serum may co‐precipitate with EVs during this process, and as a result, we cannot completely rule out the possibility that these particles contain lipoproteins or other non‐EV components. While we followed standard protocols for EV isolation, we cannot definitively attribute these particles to endosomal pathways. Future studies may benefit from employing more refined isolation techniques or combining different analytical methods to further validate the source of these particles. Nevertheless, our study provides valuable insights into the role of EVs in TNBC progression, particularly highlighting the potential role of ITGB2 in this process. Our results suggest that EVs, through their specific molecular cargo, particularly ITGB2, may play a key role in promoting the activation of CAFs. However, further investigations are needed to clarify whether other components, such as lipoproteins, present in the EVs, influence cell‐stroma interactions within the tumor microenvironment. Secondly, our in vivo experiments were conducted using a limited number of TNBC cell lines, and the results may not fully represent the heterogeneity of TNBC. Additionally, the exact mechanisms by which ITGB2 modulates immune responses in the TME remain to be elucidated. Future studies should focus on expanding the range of TNBC models studied, exploring ITGB2's interaction with other immune checkpoints, and validating ITGB2 as a therapeutic target in preclinical and clinical settings.

## 4 Conclusion

In conclusion, our study highlights the importance of ITGB2 in TNBC progression and its potential as a prognostic biomarker and therapeutic target. The overexpression of ITGB2 in TNBC tissues and serum EVs is linked to poorer patient outcomes, and its role in CAF activation and immune modulation suggests that targeting ITGB2 could disrupt key tumor‐stroma interactions and enhance the effectiveness of existing therapies, but it is likely that other EV‐derived messengers also contribute to this process. Further research is needed to explore the full spectrum of cargoes carried by EVs and their combined effects on fibroblast activation in the tumor microenvironment.

## 5 Experimental Section

### Human Tissue Samples

Tumor and adjacent tissue samples were obtained from 200 TNBC patients treated at the Affiliated Tumor Hospital of Xinjiang Medical University (Urumqi, China) between August 2017 and January 2019. All enrolled patients were pathologically confirmed to have TNBC. Demographic, clinicopathological, and survival data were collected from patient records and follow‐up examinations. Eight healthy individuals who underwent physical examination at the same hospital during the same period served as controls. This study was authorized by the Ethics Committee of the Affiliated Cancer Hospital of Xinjiang Medical University in accordance with the Declaration of Helsinki (Approval Number: 2018BC013), and all informed consent was obtained from all subjects and/or their legal guardian(s).

### Human Serum Sample Collection

Peripheral venous blood samples (8 mL each) were collected in serum (clot activator) tubes from TNBC patients (n = 3) and Luminal A, Luminal B, or Her2+ breast cancer patients (*n* = 3 each) after overnight fasting. Samples were allowed to clot at room temperature and then centrifuged at 4000 rpm for 10 min. The supernatant (serum) was collected and stored at ‐80 °C until analysis. Control serum samples were obtained from healthy subjects (*n* = 3) during physical examinations. All blood samples were free of hemolysis, hyperbilirubinemia, or hyperlipidemia, and no fibrin clots were present in the serum samples.

### Isolation and Characterization of Human Serum EVs

EVs were isolated from human serum samples using the exoEasy Maxi Kit (Qiagen, Germany) according to the manufacturer's instructions. Although this method is widely used to isolate EVs, it is important to note that the process may also co‐precipitate lipoproteins, which are commonly present in serum. Therefore, while the particles isolated in this study are referred to as EVs, we cannot definitively attribute them to endosomal or other specific cellular pathways. The presence of lipoproteins and other non‐EV contaminants cannot be entirely excluded, which may influence some of the results related to the cargo within the EVs. Freshly isolated EVs were analyzed by nanoparticle tracking analysis (NTA) using a NanoSight NS300 instrument (Malvern Panalytical, UK) to determine particle size. EV samples were stored at ‐80 °C for further analysis. EV surface markers CD63, CD9, and CD81 were detected by Western blot analysis. Briefly, EVs were lysed in RIPA buffer containing PMSF at 4 °C for 30 min and centrifuged at 12000 rpm at 4 °C for 10 min. The supernatant was collected, and protein concentration was measured using the BCA assay. Proteins were separated by SDS‐PAGE and transferred to PVDF membranes. After blocking with 5% non‐fat milk, the membranes were incubated overnight at 4 °C with antibodies against CD63 (ab134045, Abcam), CD9 (ab92726, Abcam), or CD81 (ab109201, Abcam). The membranes were then washed and incubated with HRP‐conjugated secondary antibodies for 1–2 h. Protein bands were visualized using ECL reagents and quantified by densitometry on a ChemiScope mini chemiluminescence imaging system (Clinx Science Instruments, Shanghai, China).

### Proteomic Analysis of Human Serum EVs

Proteomic analysis of human serum EV samples (200 µg each) was performed using the iTRAQ Reagent – 8PLEX Multiplex Kit (AB SCIEX). Labeled samples were separated by capillary HPLC on an Easy nLC system (Thermo Fisher) and analyzed by mass spectrometry on a Q‐Exactive Orbitrap mass spectrometer (Thermo Fisher Scientific). EV proteins were identified using Mascot 2.2 based on the mass spectrometry data. Proteome Discoverer 1.4 was used to quantify peptide fragment ion intensities and identify principal components. Differences in protein levels between groups were interpreted using t‐tests. Unsupervised hierarchical clustering analysis was used to assess the distribution of differentially expressed proteins (DEPs). GO and pathway enrichment analyses were performed on DEPs to identify cellular functions potentially related to TNBC development.

### Meta‐Analysis of Gene Expression from GEO Database

Raw RNA‐seq data for human TNBC and paired adjacent tissues were downloaded from the NCBI GEO database (GSE176078, GSE177043, and GSE58135). Differential expression analysis (fold change and P‐value) was performed using the DEseq package in R software to identify differentially expressed genes (DEGs). The identified DEGs were uploaded to DAVID software for GO and KEGG pathway enrichment analyses. CIBERSORTx was used to estimate the proportions of stromal and immune cells based on gene expression profiles.

### Immunohistochemistry (IHC) Staining of ITGB2 in Human TNBC and Adjacent Normal Tissues

ITGB2 expression in tumor and adjacent normal tissues from 200 TNBC patients was assessed by IHC staining, with sample conditions evaluated blindly by two independent pathologists. Staining intensity was calculated from data obtained from five randomly selected fields per sample at 10×20 magnification. The staining intensity of individual cells was graded as follows: 0, negative; 1, light yellow; 2, medium yellow or brown on a negative background, or dark brown on a light brown background; 3, dark brown on a negative background. The percentage of positive cells was estimated using a 10×4 microscope and classified as follows: 0, 0%; 1, 0–25%; 2, 25–50%; 3, 50–75%; 4, >75%. The total staining score was calculated as the sum of the staining intensity and the percentage of positive cells and classified as follows: 0, negative; 1–3, weakly positive; 4–5, positive; 6–7, strongly positive.

### Cell Culture

MDA‐MB‐231 human TNBC cell lines and MCF‐10A human mammary epithelial cell lines were purchased from Fenghui Biotechnology Co., Ltd. (Beijing, China). MCR‐5 normal human fetal fibroblast cells were purchased from Procell (Wuhan, China). MDA‐MB‐231 and MCF‐10A cells were cultured in DMEM‐high glucose (Gibco) with 10% fetal bovine serum (FBS). MCR‐5 cells were cultured in MEM with non‐essential amino acids (Gibco), 10% FBS, and 1% Pen‐Strep. All cell cultures were maintained in a humidified environment at 37 °C and 5% CO2.

### Western Blot Analysis of ITGB2 Protein Levels

Tissues, cells, and EVs were lysed in RIPA buffer containing PMSF, and centrifuged at 12000 rpm at 4 °C for 10 min. The supernatant was collected, and protein concentration was measured using the BCA assay. Western blot analysis was performed using anti‐ITGB2 antibody (ab185723, Abcam) to determine ITGB2 protein levels.

### qRT‐PCR Detection of ITGB2 mRNA Levels

Total RNA was extracted using Trizol reagent and reverse‐transcribed using the TransScript RT/RI enzyme mix (ABM, Germany). ITGB2 mRNA levels were measured by real‐time quantitative PCR using SYBR Green on a Real Time PCR system (Applied Biosystems, USA). Primer sequences of ITGB2 mRNA: Forward primer sequences (5′‐3′): TTCGGGTCCTTCGTGGACA; Reverse primer sequences (5′‐3′): ACTGGTTGGAGTTGTTGGTCA. Data were normalized to GAPDH.

### Lentiviral ITGB2 Overexpression and Knockdown

MDA‐MB‐231 cells were transfected with lentiviral vectors expressing human ITGB2 or empty vectors as controls to achieve ITGB2 overexpression. For ITGB2 knockdown, cells were transfected with lentiviral vectors expressing ITGB2‐targeting shRNA or scrambled control shRNA. sh‐NC: GAACAAGATGAAGAGCACCAA; ITGB2‐shRNA: CGTAAGAGGACGACCAGTAGA. All lentiviral expression systems were purchased from Shanghai Genechem Co., Ltd. (Shanghai, China). Overexpression and knockdown were confirmed by Western blot analysis.

### Laser Confocal Fluorescence Microscopy for EV Uptake

EVs were isolated from wild‐type or transfected MDA‐MB‐231 cells using the exoEasy Maxi Kit (Qiagen, Germany) according to the manufacturer's instructions. EVs (50 µg mL^−1^) were labeled with CM‐Dil (1.5 µg mL^−1^) at room temperature for 1 h, then collected by centrifugation at 1000 g for 30 min. MCR‐5 cells were seeded in 24‐well plates (1×10^4^ cells per well) and treated with labeled EVs (50 µg mL^−1^) at 37 °C for 24 h. Untreated cells served as controls. After treatment, cell nuclei were stained with DAPI, and EV uptake was detected by laser confocal fluorescence microscopy (LSM 700, Carl Zeiss, Shanghai, China).

### Cell Migration Assay

Log‐phase MCR‐5 cells were seeded in 6‐well plates (5×10^5^ cells per well) and grown to 80% confluence. A wound was created using a 200 µL pipette tip, and cells were washed repeatedly with PBS to remove detached cells. Cells were then treated with EVs (50 µg mL^−1^) from wild‐type or transfected MDA‐MB‐231 cells at 37 °C for 24 h. Untreated cells served as controls. After treatment, the wound area was photographed, and the wound width was measured.

### Cell Invasion Assay

MCR‐5 cells (2×10^5^) in 100 µL of serum‐free medium were loaded into the upper chamber of Transwell inserts coated with Matrigel. The lower chamber was filled with 500 µL of medium containing 10% FBS. Cells were allowed to migrate at 37 °C for 24 h. Cells remaining in the upper chamber were gently removed with a cotton swab. Cells that migrated to the lower surface of the membrane were fixed with paraformaldehyde and stained with crystal violet for 30 min. After washing with PBS, cells in 10 randomly selected fields per well were counted, and the average cell number was calculated as an indicator of cell invasiveness.

### Human TNBC Nude Mouse Model

Fifty SPF (specific pathogen‐free) BALB/c nude mice (4–6 weeks old) were purchased from the Animal Center of Xinjiang Medical University. Animals were housed in a sterile environment with controlled temperature. This study was approved by the Institutional Animal Care and Use Committee of Xinjiang Medical University (Approval NO: IACUC‐20210302‐44). Mice were randomly divided into four groups (10 mice per group) and subcutaneously injected with wild‐type, ITGB2‐overexpressing, ITGB2‐knockdown, or vector control MDA‐MB‐231 cells (1×10^7^ cells, 200 µL PBS) into the left mammary fat pad. The maximum longitudinal diameter (length, a) and maximum transverse diameter (width, b) of tumors were measured every three days with a caliper. Tumors were harvested and photographed.

### Detection of Fibroblast Activation Markers

MCR‐5 cells (after 24 h treatment with MDA‐MB‐231‐derived EVs) and mouse tumor tissues were lysed in RIPA buffer containing PMSF. Western blot analysis was performed to determine the levels of α‐SMA, FAP, and FSP using antibodies against α‐SMA (bs‐10196R; Beijing Biosynthesis Biotechnology Co., Ltd., Beijing, China), FAP, and FSP (BA1471‐2; Wuhan Boster Biological Technology Co., Ltd., Wuhan, China).

### Statistical Analysis

All data are presented as mean ± SD from at least three independent experiments. Statistical analysis was performed using SPSS version 21.0 or GraphPad Prism version 8.01 software. Quantitative variables were compared using Student's t‐test or Mann‐Whitney U test, as appropriate. Qualitative variables were analyzed using Pearson's χ2 test or Fisher's exact test. Kaplan‐Meier survival curves were generated, and differences were compared using the log‐rank test. A P‐value of less than 0.05 was considered statistically significant.

## Conflict of Interest

The authors declare no conflict of interest.

## Data Availability

The data that support the findings of this study are available from the corresponding author upon reasonable request.

## References

[1] H. Sung , J. Ferlay , R. L. Siegel , M. Laversanne , I. Soerjomataram , A. Jemal , F. Bray , Ca‐Cancer J. Clin. 2021, 71, 209. 33538338 10.3322/caac.21660

[2] Z. Cheng , Y. Luo , Y. Zhang , Y. Wang , Y. Chen , Y. Xu , H. Peng , G. Zhang , Ann. Hematol. 2020, 99, 1561. 32451710 10.1007/s00277-020-04000-x

[3] C. M. Perou , T. Sørlie , M. B. Eisen , M. van de Rijn , S. S. Jeffrey , C. A. Rees , J. R. Pollack , D. T. Ross , H. Johnsen , L. A. Akslen , O. Fluge , A. Pergamenschikov , C. Williams , S. X. Zhu , P. E. Lønning , A. L. Børresen‐Dale , P. O. Brown , D. Botstein , Nature 2000, 406, 747. 10963602 10.1038/35021093

[4] R. M. Hallett , A. Dvorkin‐Gheva , A. Bane , J. A. Hassell , Sci. Rep. 2012, 2, 227. 22355741 10.1038/srep00227 PMC3259129

[5] M. Bonotto , L. Gerratana , E. Poletto , P. Driol , M. Giangreco , S. Russo , A. M. Minisini , C. Andreetta , M. Mansutti , F. E. Pisa , G. Fasola , F. Puglisi , Oncologist 2014, 19, 608. 24794159 10.1634/theoncologist.2014-0002 PMC4041678

[6] M. Zhang , F. Zhang , J. Wang , Q. Liang , W. Zhou , J. Liu , J. Transl. Med. 2024, 22, 423. 38704606 10.1186/s12967-024-05237-0 PMC11070106

[7] L. Yin , S. Xie , Y. Chen , W. Li , X. Jiang , H. Li , J. Li , Z. Wu , X. Xiao , G. Zhang , Z. Cheng , H. Peng , Ann. Hematol. 2021, 100, 2229. 34228147 10.1007/s00277-021-04562-4

[8] H. Zhang , T. Xia , Z. Xia , H. Zhou , Z. Li , W. Wang , X. Zhai , B. Jin , Cell. Mol. Life Sci. 2024, 81, 96. 38372748 10.1007/s00018-024-05114-5 PMC10876760

[9] N. M. Anderson , M. C. Simon , Curr. Biol. 2020, 30, R921. 32810447 10.1016/j.cub.2020.06.081 PMC8194051

[10] R. Rimal , P. Desai , R. Daware , A. Hosseinnejad , J. Prakash , T. Lammers , S. Singh , Adv. Drug Delivery Rev. 2022, 189, 114504. 10.1016/j.addr.2022.114504 35998825

[11] J. A. Welsh , D. C. I. Goberdhan , L. O'Driscoll , E. I. Buzas , C. Blenkiron , B. Bussolati , H. Cai , D. Di Vizio , T. A. P. Driedonks , U. Erdbrügger , J. M. Falcon‐Perez , Q. L. Fu , A. F. Hill , M. Lenassi , S. K. Lim , M. G. Mahoney , S. Mohanty , A. Möller , R. Nieuwland , T. Ochiya , K. W. Witwer , J. Extracell. Vesicles 2024, 13, e12404.38326288

[12] Y. Chen , Y. Zhang , Z. Wang , Y. Wang , Y. Luo , N. Sun , S. Zheng , W. Yan , X. Xiao , S. Liu , J. Li , H. Peng , Y. Xu , G. Hu , Z. Cheng , G. Zhang , Cell Death Dis. 2022, 13, 586. 35798703 10.1038/s41419-022-05035-w PMC9263130

[13] H. Zhang , Y. Gao , J. Ying , H. Yu , R. Guo , J. Xiong , H. Jiang , J. Cosmet. Dermatol. 2023, 22, 2071. 36847708 10.1111/jocd.15683

[14] S. Dasetty , T. C. Bidone , A. L. Ferguson , Biophys. J. 2024, 123, 2716. 38098231 10.1016/j.bpj.2023.12.009 PMC11393677

[15] Z. Zheng , Y. Guo , Y. Zheng , H. Wu , Theriogenology 2024, 214, 307. 37956579 10.1016/j.theriogenology.2023.11.003

[16] M. Zhang , Q. Sun , Z. Han , X. Qin , T. Gao , Y. Xu , S. Han , Y. Zhang , Q. Liang , Z. Guo , J. Liu , Heliyon 2024, 10, e30877. 38774325 10.1016/j.heliyon.2024.e30877 PMC11107247

[17] H. Zhang , Y. Shi , J. Ying , Y. Chen , R. Guo , X. Zhao , L. Jia , J. Xiong , F. Jiang , Front. Endocrinol. 2023, 14, 1164692. 10.3389/fendo.2023.1164692 PMC10158729 37152956

[18] G. Shen , Q. Wang , Z. Li , J. Xie , X. Han , Z. Wei , P. Zhang , S. Zhao , X. Wang , X. Huang , M. Xu , Int. J. Biol. Sci. 2024, 20, 4799. 39309440 10.7150/ijbs.96338 PMC11414386

[19] N. Jabbari , E. Akbariazar , M. Feqhhi , R. Rahbarghazi , J. Rezaie , J. Cell. Physiol. 2020, 235, 6345. 32216070 10.1002/jcp.29668

[20] J. S. Desgrosellier , D. A. Cheresh , Nat. Rev. Cancer 2010, 10, 9. 20029421 10.1038/nrc2748 PMC4383089

[21] M. Paolillo , S. Schinelli , Cancers 2017, 9, 95. 28933725 10.3390/cancers9080095 PMC5575598

[22] Y. Liu , Y. Chen , F. Wang , J. Lin , X. Tan , C. Chen , L. L. Wu , X. Zhang , Y. Wang , Y. Shi , X. Yan , K. Zhao , Discovery Oncol. 2023, 14, 161. 10.1007/s12672-023-00765-5 PMC10465474 37642765

[23] H. Zhang , L. Jia , R. Guo , J. Xiong , H. Jiang , Gland Surg. 2023, 12, 354. 37057044 10.21037/gs-22-499 PMC10086776

[24] A. M. Newman , C. B. Steen , C. L. Liu , A. J. Gentles , A. A. Chaudhuri , F. Scherer , M. S. Khodadoust , M. S. Esfahani , B. A. Luca , D. Steiner , M. Diehn , A. A. Alizadeh , Nat. Biotechnol. 2019, 37, 773. 31061481 10.1038/s41587-019-0114-2 PMC6610714

[25] M. K. Malone , K. Smrekar , S. Park , B. Blakely , A. Walter , N. Nasta , J. Park , M. Considine , L. V. Danilova , N. B. Pandey , E. J. Fertig , A. S. Popel , K. Jin , Cancer Biol. Ther. 2020, 21, 560. 32213106 10.1080/15384047.2020.1739484 PMC7515526

[26] W. Yang , Z. Qiu , J. Zhang , X. Zhi , L. Yang , M. Qiu , L. Zhao , T. Wang , Front. Genet. 2022, 13, 878658. 35432487 10.3389/fgene.2022.878658 PMC9008733

[27] Y. Liu , S. Zhao , Y. Chen , W. Ma , S. Lu , L. He , J. Chen , X. Chen , X. Zhang , Y. Shi , X. Jiang , K. Zhao , Cell Oncol. 2023, 46, 1791. 10.1007/s13402-023-00844-3 PMC12974705 37646965

[28] Q. Wang , H. Wang , Y. Ding , M. Wan , M. Xu , Front. Oncol. 2022, 12, 926230. 35875143 10.3389/fonc.2022.926230 PMC9305334

[29] G. Mucciolo , W. Li , G. Biffi , Cancer Res. 2024, 84, 2938. 39279382 10.1158/0008-5472.CAN-24-2448

[30] M. A. Arnaout , Blood 1990, 75, 1037. 1968349

[31] Y. Sun , D. R. Mauerhan , P. R. Honeycutt , J. S. Kneisl , J. H. Norton , E. N. Hanley Jr. , H. E. Gruber , BMC Musculoskeletal Disord. 2010, 11, 19. 10.1186/1471-2474-11-19 PMC2828422 20109188

[32] F. Zhang , Z. Wu , S. Sun , Y. Fu , Y. Chen , J. Liu , Heliyon 2023, 9, e20612. 37842561 10.1016/j.heliyon.2023.e20612 PMC10570589

[33] H. Zhang , S. Tang , E. Biskup , Y. Zhang , L. Yong , L. Chen , F. Cai , Technol. Cancer Res. Treat. 2022, 21, 15330338221121086. 36000314 10.1177/15330338221121086 PMC9425899

[34] C. Su , Z. Lin , Y. Cui , J. C. Cai , J. Hou , Cancers 2022, 14, 4713. 36230637 10.3390/cancers14194713 PMC9564376

[35] H. Xu , A. Zhang , X. Han , Y. Li , Z. Zhang , L. Song , W. Wang , M. Lou , Cancer Immunol. Immunother. 2022, 71, 645. 34313821 10.1007/s00262-021-03022-2 PMC10992927

[36] R. Wang , X. Du , Y. Zhi , J. Comput. Biol. 2020, 27, 1104. 31725318 10.1089/cmb.2019.0235

[37] Z. Wu , Y. Chen , G. Yu , Y. Ma , Asian J. Surg. 2024, 47, 2939. 38431480 10.1016/j.asjsur.2024.02.106

[38] M. Zhang , F. X. Zhang , X. L. Yang , Q. Liang , J. Liu , W. B. Zhou , Transl. Oncol. 2024, 47, 102012. 38889521 10.1016/j.tranon.2024.102012 PMC11231535

[39] J. Wei , X. J. Huang , Y. Huang , M. Y. Xiong , X. Y. Yao , Z. N. Huang , S. N. Li , W. J. Zhou , D. L. Fang , D. H. Deng , P. Cheng , Ann. Transl. Med. 2021, 9, 1386. 34733938 10.21037/atm-21-3641 PMC8506550

[40] N. K. Verma , D. Kelleher , J. Immunol. 2017, 199, 1213. 28784685 10.4049/jimmunol.1700495

[41] R. F. Todd 3rd , J. Clin. Invest. 1996, 98, 1. 8690779 10.1172/JCI118752 PMC507390

[42] J. Li , Z. Wu , Y. Pan , Y. Chen , J. Chu , Y. Cong , Q. Fang , J. Cancer 2024, 15, 4072. 38947394 10.7150/jca.95339 PMC11212074

[43] S. Tang , Y. Zhang , X. Lin , H. Wang , L. Yong , H. Zhang , F. Cai , Oncol. Lett. 2022, 24, 285. 35814828 10.3892/ol.2022.13405 PMC9260715

[44] T. K. Kishimoto , N. Hollander , T. M. Roberts , D. C. Anderson , T. A. Springer , Cell 1987, 50, 193. 3594570 10.1016/0092-8674(87)90215-7

[45] A. Benedicto , J. Marquez , A. Herrero , E. Olaso , E. Kolaczkowska , B. Arteta , BMC Cancer 2017, 17, 827. 29207960 10.1186/s12885-017-3823-2 PMC5718006

[46] M. Liu , L. Gou , J. Xia , Q. Wan , Y. Jiang , S. Sun , M. Tang , T. He , Y. Zhang , Int. J. Mol. Sci. 2018, 19, 1866. 29941860 10.3390/ijms19071866 PMC6073814

[47] T. Shapaer , Y. Chen , Y. Pan , Z. Wu , T. Tang , Z. Zhao , X. Zeng , Discovery Oncol. 2024, 15, 446. 10.1007/s12672-024-01321-5 PMC11401830 39276259

[48] D. Wu , Y. Liu , J. Liu , L. Ma , X. Tong , Sci. Rep. 2024, 14, 17460. 39075165 10.1038/s41598-024-68111-5 PMC11286868

[49] X. Zhang , Y. Dong , M. Zhao , L. Ding , X. Yang , Y. Jing , Y. Song , S. Chen , Q. Hu , Y. Ni , Theranostics 2020, 10, 12044. 33204328 10.7150/thno.47901 PMC7667693

[50] R. Ribeiro , M. J. Carvalho , J. Goncalves , J. N. Moreira , Front. Mol. Biosci. 2022, 9, 903065. 36060249 10.3389/fmolb.2022.903065 PMC9437219

[51] E. Agostinetto , A. Losurdo , G. Nader‐Marta , A. Santoro , K. Punie , R. Barroso , L. Popovic , C. Solinas , M. Kok , E. de Azambuja , M. Lambertini , Expert Opin. Invest. Drugs 2022, 31, 567. 10.1080/13543784.2022.2049232 35240902

[52] P. Shayimu , M. Awula , C. Y. Wang , R. Jiapaer , Y. P. Pan , Z. M. Wu , Y. Chen , Z. L. Zhao , World J. Gastrointest. Surg. 2024, 16, 3142. 39575267 10.4240/wjgs.v16.i10.3142 PMC11577407

[53] S. Hua , W. Wang , Z. Yao , J. Gu , H. Zhang , J. Zhu , Z. Xie , H. Jiang , J. Cancer Res. Clin. Oncol. 2024, 150, 40. 38279987 10.1007/s00432-023-05580-7 PMC10822006

[54] Z. Wu , Y. Chen , D. Jiang , Y. Pan , T. Tang , Y. Ma , T. Shapaer , Discovery Oncol. 2024, 15, 785. 10.1007/s12672-024-01690-x PMC11655928 39692950

